# Hospital and Institutional News

**Published:** 1915-03-27

**Authors:** 


### HOSPITAL AND INSTITUTIONAL NEWS.

### THE 1915 "BURDETT."

WITH commendable promptitude, as compared with issues some years ago, the 1915 edition of "Burdett's Hospitals and Charities" is now published. The new volume, on whose established claims to respect it is now superfluous to enlarge, contains certain new features of importance. Particulars concerning institutions which have made no returns for the last five years are omitted, and against the name of such institution appears the statement, "No information obtainable"—a self-inflicted pillory which it is extraordinary that any respectable institution should allow itself to endure. The main new feature, however, is a chapter on the future of the hospitals, which contains a forecast of many possible changes in connection with the relief and treatment of the sick, in which questions arising out of the National Insurance Act and taxation are dealt with. To make room for this the 1915 volume does not contain the historical information comprised in the Silver Jubilee volume of "Burdett" last year, the twenty-fifth annual issue of which is thus marked as a work of reference of unique value. Those who still do not possess a copy should lose no time in procuring it, as only a few are now available. The institutions under the control of the Metropolitan Asylums Board are grouped in the new volume under a separate section, which has allowed further particulars to be added. It is also noteworthy that in the Colonial and Indian Hospitals section the name of the Inspector-General of each province, wherever obtainable, has been added, together with the date of the latest Blue-book or report issued by the authorities. As far as improvement is possible in a volume, a classic among reference works, these changes secure it, and we welcome the latest issue of this invaluable work, which is at once a history, statistical summary, and general directory of its subject. The price is, as usual, half a guinea.

### A PLEA FOR THE MOBILISATION OF HOSPITALS AND NURSES.

Now that comprehensive steps are being taken to increase very largely the number of beds available for the wounded, the problem of securing a sufficient nursing staff, as well as adequate building accommodation, is calling for solution. In this connection Lord Knutsford has suggested that all committees of hospitals should at once take in probationers and begin their training. He states that the London Hospital has already taken in about seventy short-time probationers, some of whom have had six months' and some three months' training, and points out that such partly trained probationers will be of service, under trained supervision, in the new hospitals to be opened. The difficulty of providing adequate sleeping accommodation is, of course, obvious, and the normal standard of accommodation may be as impracticable of attainment as normal conditions of work in other occupations directly dependent on the war. He makes the following suggestions for dealing with the general question:—Wherever possible short-time probationers should be taken in, who must be taught to realise that nursing in this country is as patriotic as nursing abroad; that the wounded should be better classified, in the sense that serious cases should be sent to one set of hospitals and slight cases to another; that the hospitals for slight cases should be staffed by those nurses who have retired from active work in their profession, that is to say, in military phraseology, by "dug-outs," assisted by probationers and Voluntary Aid Detachment members; that all hospitals treating serious cases should have relief hospitals attached to them, for semi-convalescent patients, so as to keep the beds free for new and serious cases. Lord Knutsford argues that if retired nurses were used to supervise probationers in the hospitals for slight cases, trained nurses, fully abreast of modern methods, would be made available for those institutions to which the more serious cases, on a better system of classification, would be sent. Such a scheme involves the mobilisation of the hospitals and of the nursing profession as a whole under a centralised system of organisation. Such a plan would hardly have seemed feasible a short time ago, but in the present emergency the need for it may become imperative.

### THE FIRST MUNICIPAL MATERNITY HOME.

THE Maternity and Child Welfare Scheme, which has been instituted by the Bradford Corporation, should prove a useful exemplar from the official praise which it has received from the lips of Mr. Herbert Samuel, who, as President of the Local Government Board, inspected the city's child welfare institutions last week. Accompanied by Mr. E. J. Smith, chairman of the health committee; Dr. Arthur Newsholme, medical officer to the Local Government Board; and Dr. J. J. Buchan, the city's medical officer of health, Mr. Samuel visited the Centre for Infant Consultations, the Leeds Road Hospital for Ear, Eye, Nose and Throat Diseases of Children, the school clinic, and the kitchen where meals for nursing mothers are prepared. After inspecting also two centres for the feeding of expectant and nursing mothers, he opened the Maternity Home in Horton Lane. This, which is established in an adapted private house, Mr. Samuel pointed out, was the first municipal maternity home to be opened in the country.

### MR. SAMUEL ON BRADFORD'S CHILD WELFARE SCHEMES.

IN an arresting speech, he remarked that the home, modest in size and cost, was of more profound significance than many a more imposing municipal building. It would serve as an example to the nation as a whole. That, he added, was why he had visited Bradford. The European war had shown the importance of numbers, and at this time the mass of a nation told. Of recent years the campaign against infant mortality had in the short period of ten years reduced the infant death-rate by nearly one-third, though it was still excessive. A high death-rate not only killed off the weak, but weakened the survivors. Recent medical researches had shown that the death-rate of infants before birth was probably at least as large as the death-rate in the first year of life among the infants that were born. The State and municipality could do much, but the infant must be saved in the main by the mother, whom these two authorities must teach. The Bradford Health Committee had realised that it must deal both with ante-natal conditions and also with the health of the children after the first year of life. The maternity home he described as an ante-natal clinic for expectant mothers, and also as a hospital where difficult cases of midwifery would be treated. He gave very high praise to the Bradford Infant Consultation Centre. He also praised the system of house visitation, and commended the proposal for another consultation centre for children up to school age. The health committee had gradually built up these institutions, and he was glad to say that the Chancellor of the Exchequer had consented to pay half of the cost of the greater part of these activities.

### A GREAT MEDICAL OFFICER OF HEALTH.

Science and humanity lose in no rhetorical sense by the death of Sir George Turner, a medical pioneer and investigator whose solid services to the Transvaal Government by his success in the prevention of rinderpest should live as long and gratefully in the memory of his countrymen as the tragic development of leprosy which supervened upon his researches into that disease. "Another Father Damien," as he was popularly described on the occasion of his knighthood, on the King's initiative, two years ago, Sir George was a Guy's man who qualified in 1872, and graduated M.B. at Cambridge, where he also took the diploma in public health. His first public health appointments were at Portsmouth and for a combined district of Hertfordshire and Essex, but the really important date in his career was 1895, when he entered the Civil Service of Cape Colony as medical officer of health. Continuing Koch's investigation of rinderpest on the latter's recall in 1897, he was placed in charge of the Kimberley rinderpest station, and at the end of five years he succeeded in stamping out the disease, which had been very prevalent. The value of his services so much impressed Cecil Rhodes that when the Government closed Dr. Turner's laboratory Mr. Rhodes provided the means to carry on the work, as there was a large demand for the serum in Rhodesia. The South African War eventually saw him supervising the concentration camps and hospitals which were being devastated by typhoid, and at the conclusion of peace he was appointed medical officer of health for the Transvaal. It was as medical superintendent of the Pretoria Leper Asylum from 1901-1907, when he retired, that he first began a close study of leprosy, which occupied all his Sundays and spare hours. It was during the researches pursued by him after his retirement in this country that he found himself a victim to the disease. His hope of important developments in the therapeutics of leprosy was not realised, but his achievement, in spite of official discouragement, was none the less a fine one, and he will remain a type and example of the pioneer medical officer of health whose services are as valuable, as they are but slowly appreciated, by his fellow-men.

### A NONAGENARIAN CHAIRMAN.

Subject to the confirmation of the subscribers at the annual meeting, which takes place at the time of our going to press, Mr. Adam F. Greenhalgh has been nominated honorary secretary to the Bolton Infirmary. The institution's committee has also had the unusual privilege of congratulating its chairman, Mr. W. Nicholson, on attaining his ninetieth birthday. As Mr. Nicholson presided at the last monthly meeting, it will be seen that he still takes an active personal interest in the conduct of its affairs, which places the hospital in what is almost certainly the unique position of having a nonagenarian chairman. At the meeting at which Mr. Nicholson presided it was decided, in response to the letter received from the British Hospitals Association, to co-operate in an application to the War Office that all voluntary hospitals be dealt with on a uniform basis in respect of the maintenance of sick and wounded soldiers. At a time when anxious questions of this importance are occupying the minds of hospital managers, it is remarkable that a man of the chairman's years should feel equal to the strain of active office, though it says much for his energies and interest that he should do so.

### THE ATTACK ON THE SCHOOLS.

The increased hospital accommodation now being arranged throughout the country shows a tendency which has already been protested against successfully at different centres, but which should not be permitted without very strong reasons. This tendency is to accommodate the wounded in schools, and to deprive the children of half their education by giving to two sections their instruction on alternate days. Apart from the fact that to disorganise our educational system for an indefinite period must be a self-condemned policy, the arguments which were adduced against the use of schools for the wounded in individual towns are in the main of universal application. In the first place, by co-operative arrangements between the local and Poor-Law authorities it should always be possible to secure institutional accommodation for the wounded by the taking over of Poor-Law infirmaries, as at Leicester, and distributing the displaced inmates among local Unions. Secondly, the amount of money required to adapt schools to hospital purposes on a large scale is necessarily great, and the result at best such a makeshift as may provide serious difficulties when the reception of many serious cases is in question. Thirdly, a valuable lesson of the war has been the success of temporary hospitals. Cambridge provides a classic instance of this, and of the semi-open-air treatment which is associated with temporary structures. Fourthly, there is, we suppose, not a single town to which large numbers of wounded are likely to be sent which does not possess a park or other public open space on which at small cost and in favourable surroundings a carefully designed hospital could be built. The relative cost would compare favourably with expensive adaptations of school buildings; and no displacement of other important functions can occur as must be the case when school buildings or local government offices are taken over for hospital purposes. We, therefore, in the interest alike of the wounded and of common sense, support the protests which are being made at Manchester and elsewhere in this connection. But this attack on the schools is becoming general, and hospital men would do well in every centre to oppose it.

### THE EPPING GUARDIANS AND THEIR MEDICAL OFFICER.

In The Hospital of February 13 we drew attention to a vote of censure passed by the Epping Board of Guardians upon the medical officer of their workhouse for ignoring the authority of the master. At a recent meeting of the Board the conduct of the medical officer was again under review. It was asserted that an urgent telephone message had been sent him at 5.30 A.M., requesting his attendance at two cases of confinement in the workhouse, and that he did not come until 8.5 A.M. After examining the master, the medical officer, and the superintendent nurse, the Guardians decided to ask for a Local Government Board inquiry. As the matter is thus *sub judice* comment upon it must be reserved. At present we must confine ourselves to pointing out that the medical officer denies that the phrasing of the telephone message was such as to suggest any urgency, and that the only regulation of the Local Government Board bearing upon the matter is that the midwife in a Poor-Law institution must summon the medical officer at once in any case in which the regulations of the Central Midwives Board require a medical practitioner to be called in.

### CLINICAL ASSISTANTS IN AN INFIRMARY.

Dr. Baly, the medical superintendent of Lambeth Infirmary, in a report to the visiting committee, states that the arrangement by which clinical assistants were obtained to augment the medical staff has now been in force six months, and had proved quite satisfactory, although he had only been able to obtain two. Both of these gentlemen would be entering for their final examination next month, and he proposed that, whatever the result, their appointment should then be terminated, unless the medical staff of the infirmary was so reduced as to render this course inadvisable and he was unable to obtain any substitutes. The Board has decided to leave the matter to the discretion of the medical superintendent.

### ANCOATS HOSPITAL, MANCHESTER.

Mr. Robert Dickinson Holt, who was for fifteen and a half years dispenser at Ancoats Hospital, Manchester, died suddenly in the dispensary on March 5. "He was," writes one who knew him, "a most conscientious and painstaking dispenser, and will be sadly missed by all those associated with his institution." He received his training at Messrs. J. Woolley, Sons and Co., Limited, wholesale druggists, Manchester. He entered as an apprentice with them in 1887 and was with them until 1893. From 1893 to 1899 he was in business at Stoke-on-Trent, and it was in the autumn of 1899 that he entered the service of Ancoats Hospital, Manchester. This post he had held successfully ever since.

### LEICESTER AS A HOSPITAL CENTRE.

It will be of interest to our readers to learn that the War Office authorities have at last agreed to enlarge the 5th Northern General Hospital by erecting accommodation for 500 additional beds. This, it will be remembered, was a course outlined in the columns of The Hospital of February 27 and March 6 last, when we urged the undesirability of using decentralised buildings, such as concert halls and schools, for hospital purposes. We are sure that the decision we now record is not only a wise one, but one which will result in a perfected administration and in less expenditure. The Mayor of Leicester, Alderman J. North, having himself been convinced as to the undesirability of using decentralised and unsuitable buildings, arranged that a deputation should wait upon the War Office and explain the advantages of the alternative scheme of enlarging the 5th Northern General Hospital. This deputation was received by Sir Alfred Keogh, the Director-General of Medical Services, and, as a result, the hospital under Lieut.-Col. Harrison's care will be at once enlarged. It is a War Office requirement that the additions shall be completed in record time. They will, therefore, be of an emergency character, and an appeal will be made to the local building industry to proceed with the work, bearing in mind the sole object of the provision of the accommodation, at the earliest possible moment. To provide still further accommodation the North Evington Infirmary has also been taken over, so that provision is now made in Leicester for the reception of some 2,000 patients.

### STEALING X-RAY APPARATUS

To steal portions of an *x*-ray apparatus from a hospital seems like stealing valuable objects from a public museum to complete a private collection; but interest in science was not alleged on behalf of the three defendants who were committed for trial last week at the West London Police Court on charges of stealing and receiving *x*-ray apparatus from various London hospitals. The first case concerned a milliampère-meter, missed from the Cancer Hospital on February 5. Evidence was given by Mr. George F. Westlake, the assistant in the special department of the hospital, and a detec tive stated that he found the instrument in a show-case, and that it was afterwards identified. Evidence also showed that losses had been experienced by four other hospitals—namely, Guy's, the Hampstead General, the London Homœopathic, and the Prince of Wales's, Tottenham. The man who was actually charged with the theft was described as an American citizen and traveller, avowed himself a medical practitioner, and admitted that he stole all the articles mentioned in the charges, and a good many others besides, which he could not remember. He stated that he did not know what he was doing when he stole them. According to the condensed reports of the proceedings no motive was apparent for the curious activities of the accused, who presumably exploited his alleged medical qualifications to secure admission to the various special departments. He also alleged that the two other defendants, described respectively as an electrical engineer and a sales manager, did not know that the goods were stolen. He added that he had dealings with them before. Hospitals have received many curious visitors in their time; and many articles have been stolen with a view to realising their value in hard cash, but it is rarely that visitors to hospitals have eyed covetously the surgical instruments or special equipment; yet a milliampère-meter, it seems, may prove irresistibly attractive.

### THE FISHING INDUSTRY AND THE HOSPITALS.

The position of hospitals on the East Coast is naturally one that commands sympathetic attention at the present time, and it is therefore interesting to learn how the Gorleston Cottage Hospital has been affected by the war. In the first place it has experienced a total loss of revenue from the fishing-boat owners and crews, which will become still more apparent next year, since many of the subscriptions are not received until January. Nevertheless donations have somewhat increased, and the financial year has ended with an increased balance. The new year which begins for the hospital this week is also noteworthy owing to the retirement of Mr. J. Durrant from the chairmanship, a post which he has held for sixteen years. Ill-health has induced him to drop some of his public duties, and his long record of service has been much appreciated. The new chairman is Mr. C. Addison-Williamson. It is remarkable that, in spite of some of the most fruitful sources of revenue having been depleted by the war, the hospital finances seem to be in such a healthy condition, and while congratulating Mr. H. F. Rogers, the secretary, we take it as a proof of our contention that the hospitals have never had a better case to present than they have to-day, or, if carefully presented, one more likely to yield results.

### THE MODERN CORONER'S COURT.

More than passing interest attaches to the first inquest lately held in the new Coroner's Court which has been provided by the Borough of Southwark, for it is an example which time will serve to spread, to the infinite gain of Coroners, juries, and public health. In presiding, Dr. F. J. Waldo, the City Coroner, wisely laid stress upon the public importance of the occasion. After praising the appointments of the Court, the mortuary, and the *post-mortem* room, he told the jury that less would be heard of objections to the useful duty of viewing the body if other authorities copied the example of the City and Southwark. Preserving apparatus made the viewing unobjectionable to the jury, infectious cases were made more safe, and identification often resulted. If only, he added, provincial and rural authorities would follow the lead given to them by London and the Coroners' Society, the dignity and usefulness of the Coroner's Court, which had been much enhanced of late years, would attain the standard due to its merits. For this purpose proper Courts, mortuaries, and *post-mortem* rooms were essential in place of the back bar or public-house parlour, with the body lying in a barn or cottage where the view or autopsy, when attempted, was made. Dr. Waldo pointed out that the formalin preserving apparatus now installed at Southwark was, except for that in use during the past seven years in the City, the only one in the British Isles. The office of Coroner is ancient and honourable, and not the least part of its dignity is derived from those services imposed on it of recent years, which in the public interest should be conducted in a proper Court and with up-to-date mortuary and other accommodation on the admirable lines now adopted at Southwark, thanks in part to the ability of Mr. A. Harrison, the Borough Engineer.

### A REST-CURE AT THE FRONT.

Special hospitals and the firing-line at first sight bear little relation to each other, but the *Morning Post* lately contained an account of a special hospital at the Front which is of interest. The institution is a convalescent hospital, not for those who have been wounded, operated upon, or dismissed after institutional treatment, but for those men whom exhaustion, over-strain, or some other cause has made temporarily unfit for duty. Organised by Lieutenant-Colonel A. L. F. Bate, R.A.M.C., commanding the 4th Stationary Hospital, to relieve ambulance trains and base hospitals of those who needed merely rest and care, the hospital takes, for a fortnight's treatment only, those who would otherwise be sent down to the base with other more permanently injured soldiers. Situated, we learn, in a jute factory, " lit electrically and by a glass roof, and heated by pipes carried in mid-air the whole length of the building," the hospital accommodates 1,000 patients. It is described as consisting of an enormous room, divided into wards and other rooms of all kinds by hung canvas screens. There are, of course, outside buildings for stores, boilers, and disinfectors, the latter, owing to the condition of the men, taking a very large part in the routine work. Treatment consists, one may almost say, of bed and breakfast. At least as much sleep as a man likes and four large meals a day form the main programme. If a fortnight of this rest and feeding up do not serve to recuperate the patient, he goes on to the base. After a few days in bed he not only eats, but plays games or writes letters, and has his feet and hair attended to. He then parades daily, and the amount of exercise is gradually increased. An ordnance depôt is attached to the hospital, which has been of much value in speeding up fitting and refitting. It is perhaps also worth placing on record that the writer states that, thanks to war, all creeds use the little chapel without sense of injury.

### THE EXTENSION OF HERTFORD COUNTY HOSPITAL.

Since our advocacy of a vigorous policy for Hertford County Hospital backed up the energetic counsels of Mr. Clinton Baker and Admiral Hastings, there has been sufficient financial response to show how correct we were in urging that there was no time like the present for raising money for the voluntary hospitals. Mr. Baker's object was to raise £1,600 to pay off an accumulated overdraft at the bank; but we pointed out that a definite object in view—such, for instance, as the reopening of the children's ward—would be more likely to excite public opinion. What has been the result? Mr. Clinton Baker, as announced at the recent annual meeting, has raised £1,547, and also secured £150 in new or increased annual subscriptions, while the shilling fund organised by the *Hertfordshire Mercury* has already reached some £600. In short, more than enough has been gained to show the value of continued efforts, which the large attendance at the annual meeting should help to sustain and extend. The Mayor, Mr. Purkiss Ginn, pointed out the need for improving the small bathrooms, in which slop-sinks are placed, and the underground kitchen. The absence of a lift, the bad nurses' quarters, the fact that all the servants were "huddled together in one room, except one who slept in a cupboard," were all indicated as grave defects which should not be allowed in a modern hospital. Of the £12,000 required to carry out alterations and additions to the hospital which were decided on a year or two ago, about £5,500 has been promised or subscribed. The remainder can be raised if the present efforts are continued. Admiral Hastings urged that the building scheme should be begun, and he moved that Part 4 of the scheme, which concerned the erection of two new wings, should be undertaken. After considerable discussion, in which Dr. Shelly forcibly urged the advantage of a new hospital, Admiral Hastings' plan was approved. Dr. Shelly instanced Bedford as an example of an institution which had rebuilt entirely under difficulties as great as those at Hertford, and pointed to the hospital at Windsor, built in 1904, which had been erected on a system of hollow terra-cotta blocks.

### THE VALUE OF THE HONORARY SECRETARY.

Since honorary secretaries of institutions, except in the case of cottage hospitals, have, as a rule, a permanent secretary on whom the bulk of the work and the regular routine must fall, it is sometimes questioned whether the office, in the larger institutions which still retain it, is not a survival of former conditions now passed away. There is a good deal to be said for this view, since the honorary secretary originally was a benevolent person who gave such time and personal service as he could spare to hospitals in their early days before the growth of the work and the modern standard of efficiency necessitated the appointment of a whole-time and highly trained official. Nevertheless, the suggested resignation of an honorary secretary to a hospital of seventy beds the other day provoked a tribute to this class of official which is worth attention. Much of the work, the speaker pointed out, could be delegated to the permanent official, but every honorary secretary whom they had had seemed to have set a personal ideal before him in respect of achieving some particular improvement in the finance, the organisation, or the equipment of the institution. This view, sincerely expressed, we have no doubt, represents the argument for the retention of an office which, it may be urged, by dividing the responsibility between a paid and unpaid official, while lessening the prospects and salary of the former, has the effect of depriving the institution of one trained administrator capable of doing the work which two probably not first-rate men are now dividing between them. Surely an ideal like that alluded to need not be the peculiarity of the honorary official, but is really the characteristic of every man under the voluntary system to-day worthy of responsible office. The honorary secretary in the larger institutions represents the transition stage between the old days, pointed to by Mr. Burford Rawlings' recent articles, when good will merely was the guarantee of efficiency, and modern times when hospital administration is a profession to itself. The eulogium quoted, however, is of interest as throwing into perspective the development of the secretarial post.

### DISABLED MEN IN THE NEW ARMY.

When the convalescent soldier is mentioned it is too commonly supposed that the problems relating to him are solved when a country-house, some medical supervision, and special comforts are provided. There is also the problem of the convalescent soldier's wife, who is looking after him on sick furlough, and is probably unable without aid to provide the extra nourishment incidental to complete the recovery of a man from accident, wounds, or operation. The two societies for the benefit of sailors and soldiers have power to make advances, and to pay 10s. a week on account of the soldier's pay; but in the case of men enlisted in the new Army and fallen ill enough to become disabled, delay is caused through the difficulty found in proving whether the sickness is entirely due to military service. Such a delay should have been unnecessary, since, if the man in the new Army is held by the Army doctors fit enough to be passed, any subsequent ailment cannot without grave injustice be imputed to any other cause but military service. The only exception to this rule is in the case of syphilis, though even here the contraction of the disease should not always be assumed to be the man's own fault.

### GERMAN TRADE-MARKS FOR DRUGS.

Since the Emergency Act came into force empowering the Board of Trade to suspend the trade-marks of alien enemies in favour of British applicants several Orders have been made by the Board, the effect of which has been to make the marks in question public property. One of the most important of these Orders is that which has just been made in respect of the word "aspirin." Hitherto it has been illegal for any but the owners of the trade-marks to sell acetyl-salicylic acid under the name "aspirin," and all sorts of fancy words have been coined to designate this drug. Now, however, the name "aspirin" may be applied to acetyl-salicylic acid from any source, and British makers of the drug will be able to sell it under the name by which it was first introduced into medicine and by which it is best known. It is to be hoped, however, that the effect of this will not be to increase the popularity of a drug which is already used too freely by people who doctor themselves.

### ABOLISHING A HOSPITAL'S CONSTITUTION FOR THE DURATION OF THE WAR.

Probably nothing could bring home more vividly to our minds the scarcity of medical men than the resolution moved last week by Mr. J. R. H. Smyth, chairman of the board of Birmingham General Hospital, authorising the board provisionally to suspend Laws 87 and 88. The effect of this suspension, which was approved, is for the time being to abolish the requirement that members of the resident staff should be registered practitioners. Mr. Smyth pointed out that it had been found impossible to fill some of the junior positions with qualified men. But opportunity was taken on the same occasion to alter still more radically the established constitution, for it was also resolved to authorise the board of management to modify, amend, or suspend temporarily any of the laws of the institution relating to the management of the hospital, for any period not exceeding the duration of the present war, in such a manner as may be considered necessary. Probably few resolutions of so sweeping a character have ever been agreed to with so little comment at any hospital meeting. The board is thus given a free hand, and is constituted, as it were, dictator. Whether circumstances have really made such a policy essential is perhaps open to question, and we would even ask if every possible expedient has been tried before the decision to open resident posts to unqualified assistants was agreed to.

### THE LEICESTER BOARD OF GUARDIANS AND THE SANITARY COMMITTEE.

The North Evington Infirmary having been taken over by the War Office, the Leicester Guardians asked the sanitary committee to undertake the treatment of all the consumptives in the infirmary. The sanitary committee offered to take the children, but refused to take the adults. The matter was discussed at a recent meeting of the Guardians, and the sanitary committee were sharply criticised for lack of patriotism and for failing to do all they could to help the Guardians. The medical officer of health has now issued a statement defending his committee. He points out that, allowing 100 square feet of floor space per patient, the sanitary committee's hospital will accommodate only fifty-three patients, whilst the Guardians wanted accommodation for sixty-eight; that the committee were under an obligation to the Insurance Committee to provide for insured persons with consumption; that the hospital was really intended for small-pox, and that the sanitary committee had undertaken to return the tubercular cases to their homes if a small-pox epidemic occurred—a removal which might be difficult in the case of Poor-Law patients. The medical officer of health's apology fails, because he does not realise that conditions are no longer normal. A pressing need has to be met and must be met in many cases by makeshift arrangements. His standard of space per bed, his caution as regards the possibilities of small-pox, would be admirable in ordinary conditions, but they are out of place when it is a question not of what is best, but of what is the best possible. It is to be hoped that the sanitary committee will reconsider the matter and will undertake the work the Guardians have requested—work which is only a small part of the burden the Guardians have cheerfully shouldered to help the War Office.

### THIS WEEK'S DRUG MARKET.

There has been no falling off in the volume of business and the general tendency of prices continues to be in an upward direction. Cod-liver oil is again dearer. Barbitone, acetanilide, sulphonal, and the salicylates are becoming still scarcer, and it is probable that the highest values have not yet been reached. Morphine and codeine are in strong demand, and the same applies to iodides and mercurials. The price of opium is not so firmly maintained. A further advance in the price of cocaine is probable. Ammonium carbonate is dearer. Bismuth salts are not unlikely to become dearer. Santonin is cheaper. Ipecacuanha has a lower tendency as supplies are more plentiful. At the public auction of drugs in Mincing Lane higher prices were paid for a number of drugs; senna, which is scarce, sold at high rates; Cape aloes was slightly dearer, as also were cascara sagrada and cascarilla.

### TO OUR READERS.

Contributions are specially invited from any of our readers to these columns. They should deal with topical subjects and news. They must be authenticated for the information of the Editor only. The minimum payment if published is 5s. There is no hard-and-fast rule as to space, but notes of about twenty lines in length are preferred.

